# Supplementary material for: The genome sequence of the Common Alderfly, Sialis lutaria (Linnaeus, 1758)
Source: Wellcome Open Res. 2024 Jun 19;9:322. [Version 1] doi: 10.12688/wellcomeopenres.22466.1 (PMC11462116; doi:10.12688/wellcomeopenres.22466.1)
Supplement: Peer review history

# Reviewer report: Reviewer response for version 1

**DOI:** 10.21956/wellcomeopenres.24754.r100370

**Published:** 2024-10-08

**Authors:** Xinglong Huang

**Affiliations:** 1 Jishou University, Jishou, Hunan, China

This paper reports the genome of the alderfly *Sialis lutaria*. This is novel and important information regarding a Megaloptera species and may prove useful bioinformatic and molecular data for future phylogenetic and molecular studies on this species.

 1. Only one specimen was used in the DNA or RNA  sequence. Does the genome sequence require biological replication?

Are sufficient details of methods and materials provided to allow replication by others?

Yes

Is the rationale for creating the dataset(s) clearly described?

Yes

Are the datasets clearly presented in a useable and accessible format?

Yes

Are the protocols appropriate and is the work technically sound?

Yes

Reviewer Expertise:

molecular biology of aquatic insects

I confirm that I have read this submission and believe that I have an appropriate level of expertise to confirm that it is of an acceptable scientific standard.

# Reviewer report: Reviewer response for version 1

**DOI:** 10.21956/wellcomeopenres.24754.r100376

**Published:** 2024-10-08

**Authors:** Yang Zhao

**Affiliations:** 1 Jiangsu academy of Agricultural sciences, Nanjing, China

1. While the background gives an overview of *Sialis lutaria* biology, the rationale for sequencing this species is not fully explained. Why is it essential to have the genome of *Sialis lutaria* specifically? The introduction could benefit from more context about the significance of this species in broader ecological or genetic research. And it is recommended to replace Figure 1 with a photograph of the sequenced specimen for greater relevance.
2. In the description of sample processing, the details on tissue extraction, especially concerning the *ikSiaLuta1* specimen, are lacking. The report does not mention if sectioning or dividing was performed before extraction, which could be important for the reproducibility of the study.
3. The report should provide more clarity regarding the options used for the software tools. A mention of the default parameters and whether any adjustments were made would be valuable to ensure clarity for readers attempting to replicate or build upon this work.
4. While mentioning the use of Pacific Biosciences HiFi sequencing, the manuscript omits specific details on the kit/version/chemistry. This information is crucial for reproducibility and should be clearly stated.
5. The manuscript does not offer much discussion on the implications of the genome sequencing results, aside from presenting assembly statistics. It would benefit from some analysis of the potential biological insights gained from this genome, such as evolutionary relationships or functional genomics perspectives, especially considering that *Sialis lutaria* is the dominant predator in some ecosystems.
6. The manuscript hints at how this genome could be used for further phylogenetic work but does not sufficiently expand on what specific evolutionary questions or hypotheses this genome could help address.

Are sufficient details of methods and materials provided to allow replication by others?

Partly

Is the rationale for creating the dataset(s) clearly described?

Yes

Are the datasets clearly presented in a useable and accessible format?

Yes

Are the protocols appropriate and is the work technically sound?

Yes

Reviewer Expertise:

Neuroptera

I confirm that I have read this submission and believe that I have an appropriate level of expertise to confirm that it is of an acceptable scientific standard, however I have significant reservations, as outlined above.

# Reviewer report: Reviewer response for version 1

**DOI:** 10.21956/wellcomeopenres.24754.r93652

**Published:** 2024-08-29

**Authors:** Mark T Gasser

**Affiliations:** 1 Johns Hopkins University Applied Physics Laboratory (Ringgold ID: 70603), Laurel, Maryland, USA

In the manuscript “The genome sequence of the Common Alderfly, *Sialis lutaria* (Linnaeus, 1758),” the authors report a genome assembly for a single *S. lutaria* larvae from the combination of PacBio HiFi and Illumina Hi-C sequencing. The resulting genome sequence is 147 contigs (55 scaffolds) totaling 462.0 M bases in length and includes an assembled mitochondrial genome of 15.59 K bases.

 Overall, the report is concise and clearly written, with informative figure legends.

**Minor comments:**

- Missing “and” between PacBio and Illumina sequencing in the sentence: “The specimen with ID NHMUK014361817 (ToLID ikSiaLuta1) was used for PacBio DNA sequencing Illumina Hi-C sequencing.”
- “Pacific Biosciences HiFi circular consensus DNA sequencing libraries were constructed according to the manufacturers’ instructions.” What kit/version/chemistry?
- “Hi-C data were also generated from head tissue of ikSiaLuta1 using the Arima v2 kit.” If the specimen ikSiaLuta1 was sectioned or divided prior to DNA extraction, those details should be included.
- Please include options executed for software and/or a statement that says, "Default parameters were used except where otherwise noted."
- Under Data availability, there is a statement about using the RNA-Seq data for annotation, but it may be beneficial to include this or something similar within the report text since it is covered within the Methods.

Are sufficient details of methods and materials provided to allow replication by others?

Yes

Is the rationale for creating the dataset(s) clearly described?

Yes

Are the datasets clearly presented in a useable and accessible format?

Yes

Are the protocols appropriate and is the work technically sound?

Yes

Reviewer Expertise:

multi-omics, systems biology, symbiosis

I confirm that I have read this submission and believe that I have an appropriate level of expertise to confirm that it is of an acceptable scientific standard.
